# Focus on Opportunities or Limitations? Their Effects on Older Workers’ Conflict Management

**DOI:** 10.3389/fpsyg.2020.571874

**Published:** 2020-11-05

**Authors:** Dannii Y. Yeung, Alvin K.-K. Ho

**Affiliations:** Psychology Laboratories, Department of Social and Behavioural Sciences, City University of Hong Kong, Kowloon Tong, Hong Kong

**Keywords:** conflict management, age differences, occupational future time perspective, focus on limitations, focus on opportunities

## Abstract

Socioemotional selectivity theory (SST) stresses that future time perspective (FTP) affects one’s goals and behaviors. In the work context, older workers’ occupational future time perspective (OFTP) also impacts their work-related behaviors. Two studies investigate whether the two components of OFTP, namely, focus on opportunities and on limitations, could account for the age differences in the use of conflict strategies at work. Study 1 comprises 416 Hong Kong Chinese workers aged between 20 and 68 years who completed an online questionnaire measuring their OFTP and habitual use of five conflict management strategies (integrating, compromising, obliging, avoiding, and dominating). In Study 2, 268 managerial employees and professionals were asked to recall a real-life workplace conflict that happened in the past six months and their use of the five conflict strategies in this incident. The results of Study 1 showed a negative indirect effect of age on all five conflict strategies through focus on opportunities, whereas a positive indirect effect of age was observed on obliging, avoiding and dominating strategies through focus on limitations. These age-related patterns are largely replicated in Study 2. These findings suggest that aging workers’ increased perception of limitations make them utilize less constructive strategies when facing conflict at work.

## Introduction

Interpersonal conflict is characterized by the situation in which disagreement between two or more interdependent parties is encountered, resulting in an experience of negative emotions (Barki and Hartwick, 2004). In the work context, the ways to handle conflicts considerably affect the relationship with co-workers (Dijkstra et al., 2009) and work-related outcomes (Lazarus, 2014). Examining how different individual factors predict working adults’ conflict management is crucial. Research from lifespan development reveals that younger and older adults exhibit different preferences for handling interpersonal conflicts (Blanchard-Fields et al., 2004; Fingerman et al., 2008; Davis et al., 2009; Yeung et al., 2015). Given the steady population growth of older workers in Hong Kong’s labor force and other developed countries, the topic regarding the age differences in conflict management at work deserves additional attention.

Socioemotional selectivity theory (SST; Carstensen et al., 1999; Carstensen, 2006) postulates that one’s goal orientation and behaviors change with age due to the increasingly limited perception of future time. Past studies have demonstrated that future time perspective (FTP) could account for the age differences in preferences for social partners (Fung et al., 1999) and use of problem-solving strategies in interpersonal contexts (Yeung et al., 2012). However, these studies treated FTP as a unidimensional construct. Cate and John (2007) identified two distinct dimensions of FTP, namely, focus on opportunities and on limitations. Given that these two dimensions change in an opposite direction with age, whether older workers’ selection of conflict management strategies is determined by their focuses on opportunities or limitations is questioned. This project intends to address this research question through two empirical studies.

### Socioemotional Selectivity Theory

According to SST (Carstensen, 2006), one’s perception of how much time is left in life influences his/her goal orientation. A person is likely to be directed towards the goals related to knowledge acquisition when he/she perceives his/her future as open-ended. Conversely, he/she is likely to be directed towards the goals related to maximizing emotional experiences when he/she perceives future time as limited. Age differences in goal orientation are thus observed as increasing age is associated with a limited FTP. Younger adults often prioritize knowledge-related goals over emotional goals as they hold an open-ended FTP whereas older adults prioritize emotional goals as they have a limited FTP. Previous experimental studies have demonstrated the impact of FTP on preferences for social partners (Fung et al., 1999), it is therefore expected that FTP can affect younger and older adults’ work-related behaviors, such as conflict management.

Future time perspective was used to be a single-factor measure. Using both cross-sectional and longitudinal data, Cate and John (2007) identified two distinct dimensions of FTP, namely, focus on opportunities and on limitations. Focus on opportunities refers to the perceived remaining time and possibilities in one’s life, whereas focus on limitations refers to the perceived constraints in one’s life. These two dimensions show different associations with age. Particularly, the level of focus on opportunities declines from young adulthood to early middle adulthood and remains stable over the rest of middle age. On the contrary, the level of focus on limitations is relatively stable in young adulthood but increases from one’s 40 s to 60 s. These two dimensions were moderately negatively correlated with each other and showed different patterns of association with personality characteristics (Cate and John, 2007). For instance, neuroticism was substantially correlated with focus on limitations, but not with focus on opportunities, whereas conscientiousness was moderately correlated with focus on opportunities but not with focus on limitations. Therefore, the present research aims to test their individual effects on conflict management in the work context.

### Age Differences in Conflict Management Strategies

Previous research has used various approaches to measure individuals’ behavioral responses to interpersonal conflict. For instance, Rahim (1983) adopted a dual-concern model using two dimensions, namely, concern for self and concern for others and identified five conflict styles (including integrating, compromising, obliging, dominating, and avoiding). Other researchers categorized conflict strategies into three distinct groups, namely, constructive, passive, and destructive strategies (Birditt and Fingerman, 2005; Birditt et al., 2005; Davis et al., 2009; Yeung et al., 2020). Constructive strategies include discussing openly with the conflict partner about the dispute or actively seeking help from outsiders. Integrating and compromising from the Rahim’s (1983) model are regarded as constructive strategies. Passive strategies refer to the attempts which could bring short-term benefits to the parties by minimizing the escalation of negative emotions during the conflict incident. Avoiding and obliging from the Rahim’s (1983) model are examples of passive strategies. Destructive strategies, such as dominating and yelling, involve the expression of anger and use of confrontation for forcing the other party to give in to one’s demand, resulting in undesirable relationship outcomes (Davis et al., 2009). When consequences of conflict strategies are taken into consideration, integrating and compromising are commonly perceived as adaptive strategies (Gross and Guerrero, 2000), whereas obliging, avoiding, and dominating are often regarded as maladaptive strategies because they are likely to bring unfavorable psychological or work-related outcomes to the employees, such as amplified strain (Dijkstra et al., 2009) and lowered team performance (Alhamali, 2019).

Studies have observed age differences in conflict strategies. Firstly, according to SST, the use of passive strategies is expected to be more evident among older adults than their younger counterparts due to their priority of emotional goals over knowledge-related goals. The use of passive strategies could help older adults to maximize their emotional satisfaction with important others. Empirical evidence lends support to this proposition, with older individuals exhibited greater use of passive strategies in the face of interpersonal tensions (Blanchard-Fields et al., 1997, 2007; Birditt and Fingerman, 2005; Birditt et al., 2005; Yeung et al., 2012). Similar age effect has also been observed in the work context (Davis et al., 2009; Yeung et al., 2020).

Secondly, older adults adopt destructive strategies in a lesser extent compared with their younger peers (Birditt and Fingerman, 2005; Birditt et al., 2005). In line with the theoretical framework of SST, the age-related decline in the use of these strategies serves as an effective regulatory means to minimize the potential negative consequences to their emotional experiences. However, such an age effect is not consistently shown in the workplace (Davis et al., 2009; Yeung et al., 2015, 2020), partly due to the nature of work conflict, such as the perceived future relationship with the conflict partner.

Lastly, the age effect on constructive strategies in the work and non-work contexts are considerably mixed. Some studies have reported an age-related increase in such strategies (e.g., Bergstrom and Nussbaum, 1996); other studies have shown an opposite pattern (e.g., Quayhagen and Quayhagen, 1982; Yeung et al., 2012) whereas some have found no association between age and these strategies (e.g., Feifel and Strack, 1989; Birditt et al., 2005). In the work setting, the age difference in constructive strategies was often absent (Davis et al., 2009; Yeung et al., 2020), suggesting that workers of all ages utilize this type of strategies similarly.

Inferring from the propositions of SST that FTP influences one’s social behaviors, it is possible that the abovementioned inconsistent age effects on conflict strategies may be due to the individual variations in focus on opportunities and on limitations. To disclose the true effect of age, the present project investigates the associations among age, the two dimensions of OFTP, and conflict management strategies.

### Occupational Future Time Perspective and Conflict Management Strategies

The theoretical concepts of SST have been widely used to justify the age differences in behavioral reactions to interpersonal tensions (e.g., Birditt et al., 2005; Davis et al., 2009), but most of these studies failed to explicitly test the effect of FTP on such associations. Using the hypothetical scenarios depicting conflicts with family members and close friends, Yeung et al. (2012) demonstrated that FTP, as a unidimensional construct, could partially account for the age-related decrease in problem-focused strategies (such as active coping and planning) when confronting with daily problems. However, their study did not find any significant mediating effect of FTP on the age-related increase in passive emotion-regulation strategies (such as behavioral disengagement and self-distraction). Whether or not the non-significant mediating effect of FTP on the age-related increases in passive strategies was due to the mixed effects from focus on limitations and on opportunities, which could not be differentiated in a single-factor measure of FTP, remains questioned. According to Cate and John (2007), recognizing one’s time in life is running out would be associated with perceptions of fewer opportunities and greater limitations, which subsequently activate the prioritization of emotional goals and lower emphasis on knowledge-related goals (Carstensen, 2006). Thus, examining the roles of focus on opportunities and on limitations would help to clarify their influences on behavioral responses to negative events (Strough et al., 2016).

To reflect working adults’ FTP in the work context, occupational future time perspective (OFTP) should be considered. OFTP has been used to examine work-related phenomena among aging workers. For instance, studies have demonstrated that focus on opportunities at work was found to be positively related to work engagement (Schmitt et al., 2013) and work performance (Zacher et al., 2010). Some researchers treated OFTP as a tri-dimensional construct, which includes perceived remaining time, focus on opportunities and on limitations (Zacher, 2013; Topa and Zacher, 2018), but the results of a recent meta-analytic study on OFTP (Rudolph et al., 2018) demonstrated that remaining time and opportunities could be combined as one dimension as they were highly correlated. Such results indeed align with Cate and John’s (2007) 2-dimension structure of FTP, including focus on opportunities and on limitations. Rudolph et al. (2018) also recommended future research to place more emphasis on the role of focus on limitations, which was largely neglected in the current literature.

Evidence demonstrates that age is negatively associated with focus on opportunities and positively associated with focus on limitations (e.g., Cate and John, 2007; Rohr et al., 2017; Rudolph et al., 2018). Therefore, it is anticipated that individuals who focus on opportunities tend to prioritize knowledge-related goals whereas individuals who focus on limitations are likely to emphasize emotional goals, which subsequently influence their behavioral responses to workplace conflict. Several studies using OFTP lend insights into how focus on opportunities and on limitations would explain the effects of age on various conflict management strategies. For example, age was found to exert a negative indirect effect on work performance through focus on opportunities, despite that no direct effect of age was found (Zacher et al., 2010). Applying the possible selves theory (Markus and Nurius, 1986), Zacher et al. (2010) explained that individuals who have a more positive anticipation about their occupational future are more motivated to strive for a better performance at work than individuals who have less positive anticipation. The use of constructive strategies in the workplace shares considerable commonalities with work performance as they could both be considered desirable work-related outcomes that bring better organizational performance. Moreover, both task and contextual job performances were found to be positively predicted by the use of constructive strategies, such as integrating (Trudel, 2009). Inferring from the previous research in which focus on opportunities has been shown to account for the age effects on desirable work-related outcomes, the following hypothesis is proposed:

H1: A negative indirect effect of age will exist on the use of constructive strategies (including integrating and compromising) through focus on opportunities.

Past studies distinguished the effect of focus on limitations on work-related behaviors from that of focus on opportunities (Zacher, 2013; Topa and Zacher, 2018). For example, Topa and Zacher (2018) found that only focus on limitations significantly predicted retirement intention, but the effect of focus on opportunities on retirement intention was not observed. Focusing on limitations motivates individuals to maximize their affective experiences and interpersonal closeness, instead of directly managing the conflict issue by utilizing constructive strategies. Given that only limited studies have examined the effect of focus on limitations in the work context (Rudolph et al., 2018), this project intends to advance the current literature to examine whether focus on limitations could explain the age-related increase in passive strategies (including obliging and avoiding) and the age-related decrease in destructive strategies. Thus, the following hypothesis is formulated:

H2: A positive indirect effect of age will exist on the use of passive strategies (including obliging and avoiding) and a negative indirect effect of age will exist on the use of destructive strategies (i.e., dominating) through focus on limitations.

### The Present Project

The present project aims to clarify the effects of age on conflict management strategies at work through the lens of the two dimensions of OFTP, namely, focus on opportunities and on limitations. The findings of the current project will advance the current literature on workplace conflict management by disclosing an underlying mechanisms accounting for the association between age and conflict strategies. Two studies were conducted to test the two hypotheses. Study 1 measured younger and older workers’ habitual tendency to use the five conflict strategies at work. Utilizing the approach of retrospective recall of a real-life work conflict, Study 2 examined and compared younger and older employees’ actual use of conflict strategies in a recent conflict incident with coworker.

## Method of Study 1

### Participants

Study 1 comprised 416 Hong Kong Chinese participants. Among them, 57.7% were females and their age ranged between 20 and 68 years (*M* = 39.13; SD = 12.42). About half of the participants were managerial employees and professionals (48.8%), and the remaining (51.2%) was from other occupations (e.g., clerical, service-oriented, or technical workers). The majority of the participants (64.9%) completed a bachelor’s degree or higher qualification and the remaining completed an associate degree or lower qualification.

### Procedure

Human ethics approval (H001009) was obtained from the Human Subjects Ethics Sub-Committee of the affiliated university. The current study was administered online using the Qualtrics platform. The participants were recruited through the alumni mailing list of the affiliated university and posted flyers on campus. Individuals who were working full-time or part-time were eligible to participate in this study. Informed consent was obtained from each participant at the beginning of the study. The duration of each questionnaire was roughly 15 min. In appreciation of his/her participation, each participant received a supermarket cash voucher worth of HKD50 (approximately US$6.5) upon completion of the online survey.

### Measures

#### Occupational Future Time Perspective

The participants’ perceived time perception in their occupation was measured using the Chinese version of the 10-item OFTP scale (Zacher and Frese, 2009; Ho and Yeung, 2016), which was adapted from the original FTP scale (Carstensen and Lang, 1996) measuring perception of future time in life. This study assessed and calculated focus on opportunities (seven items) and focus on limitations (three items) following Cate and John’s (2007) two-dimension categorization. The sample items for these two subscales are “Many opportunities await me in my occupational future” and “I have the sense that my occupational time is running out,” respectively. Each item was rated on a five-point Likert scale ranging from 1 (*strongly disagree*) to 5 (*strongly agree*). Higher scores on focus on opportunities represent that participants perceive more opportunities for future development and growth in their occupation, whereas higher scores on focus on limitations represent that participants perceive greater constraints and limitation in their occupation. The Cronbach’s alphas of focus on opportunities and on limitations in the current study were 0.88 and 0.80, respectively.

#### Conflict Management Strategies

The participants’ habitual use of conflict management strategies in the work context was assessed by the Chinese version of Rahim Organizational Conflict Inventory-II (ROCI-II; Rahim, 1983; Yeung et al., 2020). Permission to use this inventory was obtained from the Center for Advanced Studies in Management. The inventory comprises 28 items measuring five strategies, including integrating, compromising, obliging, avoiding, and dominating. The participants were asked to indicate their typical ways of addressing conflicts or disputes with their co-workers. Each item was rated on a five-point Likert scale ranging from 1 (*strongly disagree*) to 5 (*strongly agree*), with higher scores representing a greater use of the strategy. A sample item from the integrating subscale is “I try to investigate an issue with my peers to find a solution acceptable to us.” Confirmatory factor analysis was performed to verify the factor structure of the Chinese version of ROCI-II. The results showed that the model fit of the five-factor structure was satisfactory, χ^2^(138) = 474.40, *p* < 0.001, CFI = 0.90, TLI = 0.87, RMSEA = 0.08, SRMR = 0.07. Previous research has also demonstrated the predictive validity of the Chinese version of ROCI-II, for instance, the use of integrating and compromising was significantly associated with negative postconflict relationship and positive emotions (Yeung et al., 2020). The Cronbach’s alphas of the integrating, compromising, obliging, avoiding, and dominating in the current study were 0.81, 0.72, 0.82, 0.70, and 0.77, respectively.

#### Demographic Variables

The participants’ age, gender (1 = male, 2 = female), occupation (1 = managerial employees and professionals, 2 = other occupations), and education level (1 = an associate degree or below, 2 = a bachelor’s degree or above) were recorded.

## Results and Discussion of Study 1

### Descriptive Analyses

The descriptive statistics of and correlation among major variables are shown in Table 1. In support of previous research, age was negatively correlated with focus on opportunities but positively correlated with focus on limitations (*r* = −0.46, and *r* = 0.49, respectively, *p* < 0.001). Education was significantly correlated with focus on opportunities (*r* = 0.26; *p* < 0.001) and on limitations (*r* = −0.33; *p* < 0.001), and obliging (*r* = 0.15; *p* = 0.002). Occupation was significantly correlated with both focus on opportunities (*r* = −0.18; *p* < 0.001) and on limitations (*r* = 0.16; *p* = 0.001). Thus, education and occupation were controlled as covariates in the mediation analyses. Gender was not correlated with the two OFTP dimensions or the five conflict strategies, thus it was excluded from the following analyses. In addition, age was negatively correlated with obliging (*r* = −0.18; *p* < 0.001) and avoiding (*r* = −0.12; *p* = 0.015).

**TABLE 1:** Descriptive statistics and correlation among major variables in Study 1 (*N* = 416).

*Variables*	*M/*%	SD	(1)	(2)	(3)	(4)	(5)	(6)	(7)	(8)	(9)	(10)
(1) Age	39.13	12.42	–									
(2) Gender (Female)	57.7%	–	−0.04	–								
(3) Education (Bachelor’s degree)	64.9%	–	−0.49***	0.02	–							
(4) Occupation (others)	51.2%	–	0.18***	0.22***	−0.40***	–						
(5) Focus on Opportunities	3.30	0.75	−0.46***	−0.06	0.26***	−0.18***	–					
(6) Focus on Limitations	3.17	0.87	0.49***	0.04	−0.33***	0.16***	−0.50***	–				
(7) Integrating	3.81	0.47	−0.02	0.02	0.07	−0.05	0.23***	−0.06	–			
(8) Compromising	3.83	0.46	0.00	0.06	0.03	−0.02	0.21***	−0.05	0.74***	–		
(9) Obliging	3.65	0.51	−0.18***	0.02	0.15**	−0.01	0.15**	−0.01	0.43***	0.48***	–	
(10) Avoiding	3.52	0.53	−0.12*	0.01	0.01	0.02	0.09	0.10*	0.21***	0.38***	0.52***	–
(11) Dominating	3.11	0.65	−0.02	−0.08	−0.05	−0.07	0.21***	0.11*	0.21***	0.15**	0.15**	0.19***

Gender was coded as 1 = male and 2 = female. Education was coded as 1 = associate degree or below and 2 = bachelor’s degree or above. Occupation was coded as 1 = managerial employees and professionals and 2 = other occupations. *p < 0.05; **p < 0.01; ***p < 0.001.

### Indirect Effect of Age on Conflict Management Strategies Through OFTP

**FIGURE 1:**
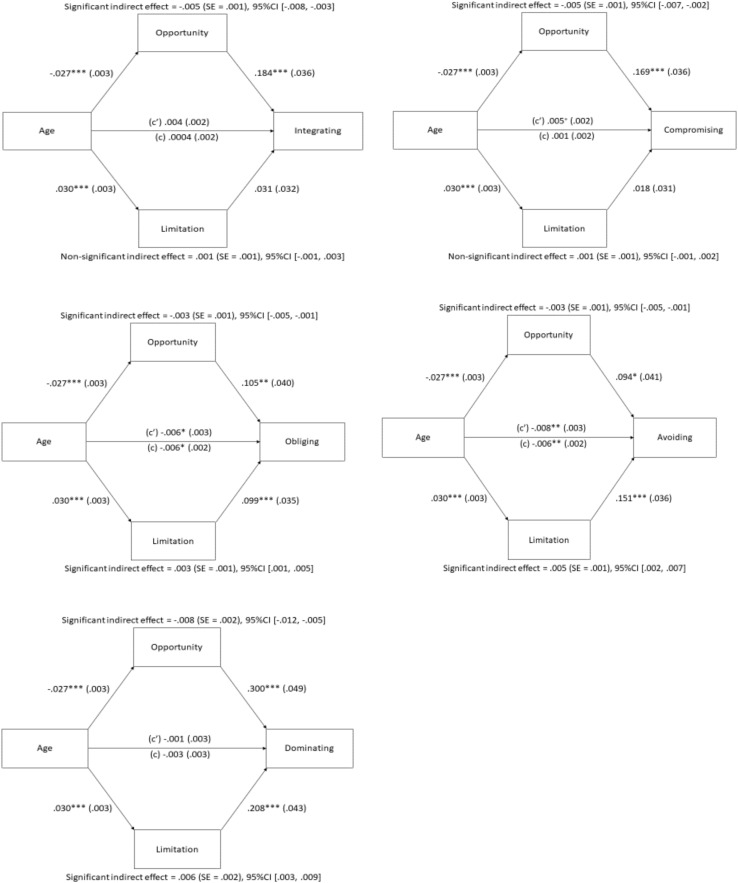
The mediating effects of focus on opportunities and focus on limitations on the five conflict management strategies measured in Study 1. *c’* = direct effect. *c* = total effect. Opportunity = Focus on opportunities. Limitation = Focus on limitations. ^∗^*p* < 0.05; ^∗∗^*p* < 0.01; ^∗∗∗^*p* < 0.001; ^+^*p* = 0.051.

To validate H1 and H2, the indirect effects of age on the five conflict management strategies through the two OFTP components were tested by the PROCESS macro (Model 4; Hayes, 2018). Focus on opportunities and focus on limitations were inputted to the model simultaneously as parallel mediators, and education and occupation were controlled as covariates as they were significantly correlated with the two dimensions of OFTP or conflict strategies. Figure 1 presents the results.

A significant negative indirect effect of age existed on all five strategies through focus on opportunities, including integrating (*B* = −0.005, SE = 0.001, 95%CI [−0.008, −0.003]), compromising (*B* = −0.005, SE = 0.001, 95%CI [−0.007, −0.002]), obliging (*B* = −0.003, SE = 0.001, 95%CI [−0.005, −0.001]), avoiding (*B* = −0.003, SE = 0.001, 95%CI [−0.005, −0.001]), and dominating (*B* = −0.008, SE = 0.002, 95%CI [−0.012, −0.005]). Consistent with H1, relative to their younger counterparts, older workers perceived fewer opportunities in their occupation, which subsequently decreased their use of constructive strategies, including integrating and compromising, for managing workplace conflicts. In addition to the hypothesized effect on constructive strategies, age also had a negative indirect effect on passive strategies (obliging and avoiding) and destructive strategies (dominating) through focus on opportunities. These results suggest that older workers’ perceived fewer occupational opportunities are associated with lower overall tendency in addressing conflict incident. This age-related effect through focus on opportunities is similar to that on work performance (Zacher et al., 2010).

A significant positive indirect effect of age on obliging (*B* = 0.003, SE = 0.001, 95%CI [0.001, 0.005]), avoiding (*B* = 0.005, SE = 0.001, 95%CI [0.002, 0.007]), and dominating (*B* = 0.006, SE = 0.002, 95%CI [0.003, 0.009]) through focus on limitations was also shown. Consistent with H2, age had a positive indirect effect on obliging and avoiding through focus on limitations. In contrast to the predicted negative indirect effect, the present study found a positive indirect effect of age on dominating through focus on limitations. As a result, H2 was partly supported.

As the indirect effects of age on obliging, avoiding, and dominating through focus on opportunities and limitations were both significant, the contrast comparisons were checked and found to be significant (Obliging: *B* = −0.006, SE = 0.002, 95%CI [−0.010, −0.002]; Avoiding: *B* = −0.007, SE = 0.002, 95%CI [−0.011, −0.003]; and Dominating: *B* = −0.014, SE = 0.003, 95%CI [−0.020, −0.010]), implying that the two indirect effects of age through focus on opportunities and limitations are different (Hayes, 2018).

The results of Study 1 demonstrate that the habitual use of conflict strategies in the workplace varies by the age of the participants, which is partly due to their perceived opportunities or limitations in their career development. However, it is worth to note that the five strategies were positively correlated with each other. Similar correlational patterns are also shown in the previous research (e.g., Aquino, 2000; Balyan, 2018). For example, Aquino (2000) found that in a culturally diverse sample of working adults (mean age = 40.7), the five strategies were all significantly and positively correlated with each other, such as dominating was positively associated with integrating and obliging. It is suspected that some of the participants in the present study might have recalled multiple conflict incidents when answering the questions of ROCI-II, thus constraining the intra-individual variability in their report of the habitual use of the five conflict strategies. For example, some participants might use obliging to a greater extent for trivial conflicts while dominating was adopted for handling conflicts that would influence their career development. As a result, positive correlations among the five conflict strategies were shown.

Moreover, compared with integrating and compromising, dominating, obliging, and avoiding are maladaptive in terms of the negative psychological or work-related consequences brought to employees. According to Rahim (1983), the five strategies vary in the levels of concern for self and concern for others. Unlike the use of integrating and compromising which bring together the concerns for both parties, the use of dominating and obliging only focuses on one of the involved parties (e.g., only concern for self: dominating, only concern for others: obliging), whereas avoiding is preferred when the employee simply wants to withdraw from the conflict situation. As a result, these three maladaptive strategies (dominating, obliging, and avoiding) are often associated with negative outcomes, such as amplified strain (Dijkstra et al., 2009) or lowered team performance (Alhamali, 2019), thus a similar result pattern was observed among these three strategies in the present study.

To get a clearer picture of the employment of various conflict strategies in the workplace, Study 2 was designed to measure younger and older employees’ actual use of conflict management strategies in a real-life conflict incident at work. With the use of retrospective recall of a specific conflict incident, it is expected that the use of contradictory strategies (such as simultaneous use of obliging and dominating) is less likely.

## Method of Study 2

### Participants

Study 2 consisted of 268 Hong Kong Chinese managerial employees and professionals. Among them, 53.7% were females and their mean age was 42.56 (SD = 9.48; Range = 23–66). The majority of the participants (75.4%) had completed a bachelor’s degree or above, and the remaining indicated that they had completed an associate degree or lower qualification. Almost 90% of the participants worked as managerial or administrative employees and the remaining were professionals (11.2%).

### Procedure

Human ethics approval was obtained from the Human Subject Ethics Committee of the affiliated university. The participants were invited through the human resources department of public and private organizations. Among the 58 organizations that responded to our invitation, 27 agreed to participate in this study (with a response rate of 46.6%) and sent out the questionnaire package to the eligible staff or members through internal mail or email. Participation was totally voluntary. The participants completed the survey themselves, and written consent was obtained on the cover page of the questionnaire. The completed survey was returned to the researchers directly to ensure confidentiality. Each participant received a supermarket cash voucher worth HKD100 (approximately US$13) as compensation for his/her participation.

### Measures

#### Personal Conflict Incident in the Workplace

To understand working adults’ behavioral responses to real-life workplace conflict, each participant was asked to recall a personal conflict incident with another coworker that occurred in the past six months. The participants were first asked to briefly describe the conflict incident by reporting the content and cause of this recalled incident as well as the gender of and relationship with the coworker involved. With reference to previous research on workplace conflict (Yeung et al., 2020), the causes of the conflict were grouped into two categories, which indicated the type of conflict reported: interpersonal (such as different viewpoints and opinions about how the task was performed, quality of work) and structural nature (such as rigid organizational rules and procedures and insufficient resources). The gender of (1 = male and 2 = female) and relationship with the coworker (1 = supervisor, 2 = peer, 3 = subordinate) were also recorded. Preliminary analyses showed that younger (aged 39 or below) and older (aged 40 or above) participants did not vary in their report of the conflict type [*X*^2^(2) = 1.45, *p* = 0.229], gender [*X*^2^(2) = 2.59, *p* = 0.270] or relationship with the conflict partner [*X*^2^(2) = 5.35, *p* = 0.069]. These three variables had no significant association with the five conflict strategies and had no effect on the mediation results reported below, thus they were excluded from the following analyses.

#### OFTP

Similar to Study 1, the two dimensions of OFTP were measured using the 10-item OFTP scale (Zacher and Frese, 2009; Ho and Yeung, 2016). Each item was rated on a five-point Likert scale ranging from 1 (*strongly disagree*) to 5 (*strongly agree*). The Cronbach’s alphas of focus on opportunities and on limitations in the current study were 0.80 and 0.68, respectively.

#### Conflict Management Strategies

The 28-item ROCI-II (Rahim, 1983; Yeung et al., 2020) was adopted to measure the extent of using the five conflict strategies in the conflict incident. Permission to use this inventory was obtained from the Center for Advanced Studies in Management. The participants were instructed to indicate whether they had used each strategy for handling the recalled conflict incident using a five-point Likert scale ranging from 1 (*strongly disagree*) to 5 (*strongly agree*). One item in the compromising subscale (“I use ‘give and take’ so that a compromise can be made”) showed poor item-total correlation, thus it was removed from this subscale. The Cronbach’s alphas of the integrating, compromising obliging, avoiding, and dominating and in the current study were 0.85, 0.59, 0.92, 0.70, and 0.82, respectively.

#### Demographic Variables

The participants’ age, gender (1 = male, 2 = female), and education (1 = an associate degree or below, 2 = a bachelor’s degree or above), and occupation (1 = managerial or administrative employees; 2 = professionals) were recorded. Preliminary analyses showed that occupation did not significantly correlate with age, OFTP subscales, or the five conflict strategies, so it was excluded from the following analyses.

## Results and Discussion of Study 2

### Descriptive Analyses

The descriptive statistics of and correlation among major variables are presented in Table 2. Gender was negatively correlated with focus on opportunities (*r* = −0.14, *p* = 0.026), whereas education was significantly correlated with focus on opportunities (*r* = 0.17; *p* = 0.006) and on limitations (*r* = −0.21; *p* < 0.001). Thus, gender and education were controlled as covariates in the mediation analyses. In addition, both age and the quadratic term of age (age^2^) were found to be unrelated with any of the five conflict management strategies, with *r* ranging from -0.02 to 0.07.

**TABLE 2:** Descriptive statistics and correlation among major variables in Study 2 (*N* = 268).

*Variables*	*M/*%	SD	(1)	(2)	(3)	(4)	(5)	(6)	(7)	(8)	(9)	(10)
(1) Age	42.56	9.48	–									
(2) Age^2^	1900.92	800.87	0.99***	–								
(3) Gender (Female)	53.7%	–	−0.21***	−0.23***	–							
(4) Education (Bachelor’s degree)	75.4%	–	−0.34***	−0.34***	0.01	–						
(5) Focus on Opportunities	3.33	0.61	−0.28***	−0.26***	−0.14*	0.17**	–					
(6) Focus on Limitations	2.94	0.77	0.42***	0.42***	0.01	−0.21***	−0.54***	–				
(7) Integrating	3.73	0.62	0.07	0.07	−0.09	−0.02	0.24***	−0.05	–			
(8) Compromising	3.67	0.59	−0.01	−0.02	0.02	−0.09	0.16**	−0.02	0.63***	–		
(9) Obliging	2.73	0.86	−0.06	−0.05	0.04	0.09	0.03	0.07	0.11	0.18**	–	
(10) Avoiding	3.15	0.64	0.01	0.01	0.03	−0.03	−0.08	0.18**	−0.14*	0.14*	0.35***	–
(11) Dominating	3.25	0.73	−0.02	−0.02	0.01	−0.03	0.05	0.09	0.17**	0.13*	−0.26***	−0.02

Gender was coded as 1 = male and 2 = female. Education was coded as 1 = associate degree or below and 2 = bachelor’s degree or above. *p < 0.05; **p < 0.01; ***p < 0.001.

### Indirect Effect of Age on Conflict Management Strategies Through OFTP

**FIGURE 2:**
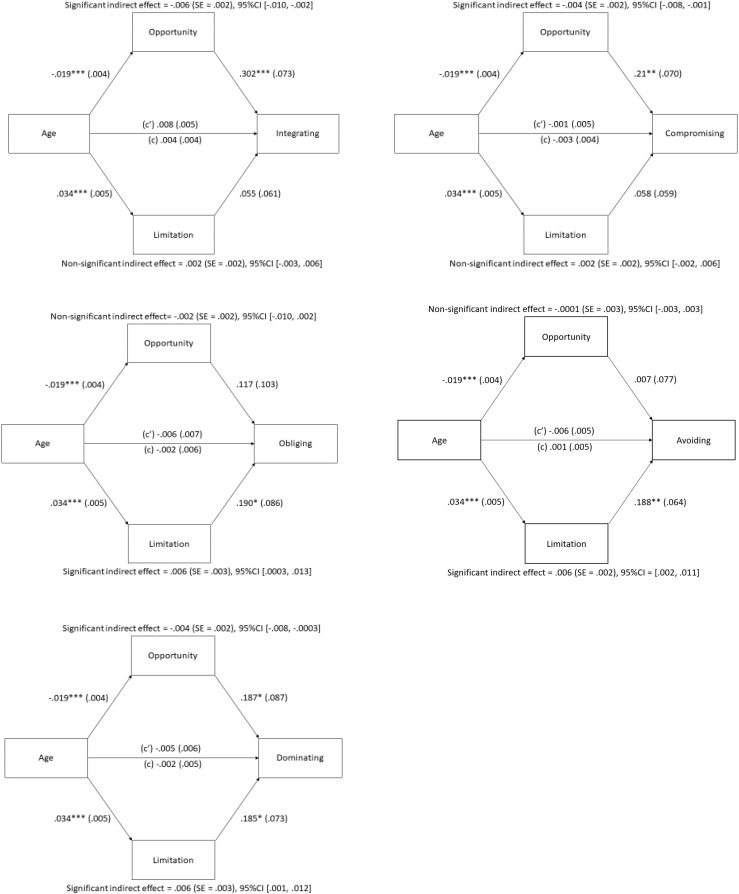
The mediating effects of focus on opportunities and focus on limitations on the five conflict management strategies measured in Study 2. *c’* = direct effect. *c* = total effect. Opportunity = Focus on opportunities. Limitation = Focus on limitations. ^∗^*p* < 0.05; ^∗∗^*p* < 0.01; ^∗∗∗^*p* < 0.001.

To test H1 and H2, the indirect effects of age on the five conflict management strategies through the two OFTP dimensions were tested by the PROCESS macro (Model 4; Hayes, 2018). Focus on opportunities and focus on limitations were inputted to the model simultaneously as parallel mediators, and gender and education were controlled as covariates. The mediation results are summarized in Figure 2.

Results indicated a significant negative indirect effect of age existed on three conflict strategies through focus on opportunities, including integrating (*B* = −0.006, SE = 0.002, 95%CI [−0.010, −0.002]), compromising (*B* = −0.004, SE = 0.002, 95%CI [−0.008, −0.001]), and dominating (*B* = −0.004, SE = 0.002, 95%CI [−0.008, −0.0003]). Moreover, a significant positive indirect effect of age existed on obliging (*B* = 0.006, SE = 0.003, 95%CI [0.0003, 0.013]), avoiding (*B* = 0.006, SE = 0.002, 95%CI [0.002, 0.011]), and dominating (*B* = 0.006, SE = 0.003, 95%CI [0.001, 0.012]) through focus on limitations. As the indirect effects of age on dominating through focus on opportunities and limitations were both significant, the contrast comparison was checked and found to be significant (*B* = −0.010, SE = 0.004, 95%CI [−0.018, −0.003]), implying that these two indirect effects are different (Hayes, 2018).

Consistent with H1, older employees perceived fewer occupational opportunities than younger employees, which was subsequently related to their fewer use of integrating and compromising strategies. In support of H2, older employees reported increased perceived limitations than their younger counterparts, which was consequently related to their greater use of obliging and avoiding strategies. Inconsistent with the prediction, the present study found a positive indirect effect of age on dominating through focus on limitations and a negative indirect effect of age on dominating through focus on opportunities. These results reveal that the age-related pattern in the use of dominating is considerably dependent on whether the employees focus on opportunities or on limitations.

## General Discussion

Building on the theoretical framework of SST (Carstensen, 2006), two studies were reported in this paper to investigate whether age indirectly predicts the use of conflict management strategies at work through the two OFTP dimensions, namely, focus on opportunities and on limitations. Inspired by Cate and John’s (2007) categorization of FTP components, it was speculated that the inconsistent age-related patterns in conflict responses shown in previous studies (e.g., Birditt et al., 2005; Davis et al., 2009; Yeung et al., 2020) might be due the distinct effects of focus on opportunities and focus on limitations. Study 1 measured younger and older employees’ habitual use of conflict strategies in the work setting, whereas Study 2 recorded their actual behavioral responses to an actual personal conflict incident via retrospection. The results of the two studies disclose that the age-related effects on the five conflict strategies vary, largely depending on whether the employees perceive opportunities or limitations in their career development.

### Focus on Opportunities and on Limitations as Mediators

Among the five strategies measured in this project, the direct effects of age were only observed in the habitual use of obliging and avoiding (see Figure 1), revealing that generally younger employees have a greater tendency to use these two passive strategies than their older counterparts when conflict arises in the workplace. These results fail to replicate the age-related increase in passive strategies shown in past studies (e.g., Davis et al., 2009; Yeung et al., 2020). One possible explanation may be related to the fact that younger workers generally have less work experiences than older workers, thus they are more likely to go along with the judgments or decisions recommended by other colleagues. This may be especially possible for Hong Kong Chinese individuals as most of the traditional virtues of collectivistic cultures, such as the emphasis of power distance and respect for elders, are largely observed at work (Aswathappa, 2010). Thus, the use of obliging and avoiding among younger employees is greater than that of their older counterparts.

However, when the two OFTP components are considered, the effect of age on obliging and avoiding becomes clear. In particular, the results of both studies reveal a positive indirect effect of age on these two passive strategies through focus on limitations. Relative to younger workers, older workers perceive greater limitations, which are subsequently associated with greater use of obliging and avoiding. This is consistent with the proposition of SST that perception of limited future time motivates the individuals to emphasize here and now and presented-oriented goals, making emotion regulation become more salient (Carstensen et al., 2003; Carstensen, 2006). Therefore, instead of actively resolving the conflict, older workers who focus on limitations prefer avoiding or obliging in handling the conflict to reduce its negative impacts on their emotional well-being. Another possible explanation for the age-related increase in utilizing passive strategies through focus on limitations may be related to the linkage between perceived limitations in the workplace and work disengagement. Work disengagement is illustrated by workers’ lack of motivation and/or interest in work and their decreased sense of loyalty to the organization (Gatchel and Schultz, 2012). Generally, older workers who are nearer to their planned retirement age are more likely to disengage from work than younger workers (Damman et al., 2013). This might be exceptionally true if one starts to foresee his/her future in the workplace is restricted (i.e., stronger emphasis on limitations at work). Therefore, older workers who are likely to be accompanied with the sense of limitedness in the workplace may choose to disengage from work by using more obliging and avoiding in the workplace. As such, these strategies are adopted more frequently by older workers with a high level of perceived limitations in the workplace despite the fact that the actual usage of these conflict strategies may lead to a negative consequence to organizational performance.

The findings of Study 1 also indicate that the age effect on obliging and avoiding becomes negative when focus on opportunities is considered. Specifically, compared with younger peers, older workers perceive fewer opportunities at work, which consequently reduces their tendency to utilize passive strategies. According to Leung (2008), adopting passive strategies at work, such as expressing deference to authority and saving face for the conflict partner, helps to maintain interpersonal harmony, which may sometimes be beneficial to one’s future career development. Ultimately, these indirect effects reveal that the tendency to utilize passive strategies is strongly tied with the working adults’ perception of opportunities or limitations in their career development.

Moreover, the results of both studies reveal that age exerts a negative indirect effect on integrating and compromising through focus on opportunities. With age, older workers perceive their occupational opportunities as diminishing over time, which subsequently reduces their use of constructive strategies to manage the conflict incident. These findings are in accordance with past studies examining the association between focus on opportunities at work and other desirable work-related behaviors and outcomes, such as work engagement (Schmitt et al., 2013) and performance (Zacher et al., 2010). These results are similar to Ho and Yeung’s (2016) study wherein the participants with a more open-ended OFTP were shown to adopt problem-focused strategies to a greater extent in the workplace than those with a more limited OFTP. Collectively, perceiving more opportunities at work motivates the employees to utilize constructive and adaptive means to resolve the conflict issue proactively because these responses are deemed beneficial to one’s career development in the long run, such as demonstrating effective problem-solving skills.

Similar to the use of obliging and avoiding, the age-related pattern in dominating is largely dependent on the individuals’ OFTP. Particularly, the utilization of dominating strategies is higher among older workers who perceive limitations at work whereas their use of these strategies is lower when they perceive opportunities of future development. These results are contradictory to the prediction that age would exert a negative indirect effect on dominating through focus on limitations. It is speculated that among older workers, the relationships with co-workers may not be valued as much as other close social relationships such as family members and close friends (Yeung et al., 2020). With an increased perception of limitations at work, older workers would put greater emphasis on their personal interests, instead of paying more attention to the mutual interests with their coworkers when a conflict occurs. As a result, dominating is more likely to be adopted by older workers whose perceived limitations in occupational development are higher.

Alternatively, these unexpected results on dominating may be explained by the heightened value of power distance among older employees with greater perceived limitations in their occupational development. Specifically, cross-cultural comparison has demonstrated that one’s use of dominating is positively predicted by his/her level of power distance (Gunkel et al., 2016). In an organization, high power distance is typically shown when older and senior employees are being respected by their colleagues mainly due to their older age and longer tenure in the company (Khatri, 2009). Given that China is well-known for its high power distance orientation (Hofstede, 1980), it is possible that older Chinese workers tend to endorse such cultural value and expect others to show greater respect and give in to their demands, thus contributing to their greater use of dominating, especially among those with increased limitations in their career development. Future studies should measure employees’ orientation of power distance to get a clear understanding of the effect of age on dominating.

### Practical Implications

Age diversity in the workforce is increasingly prevalent, thus it is important for employers and managers to realize the age variations in conflict management between younger and older workers and understand its underlying mechanism. The results of the present two studies reveal that focus on opportunities is associated with constructive and adaptive strategies such as integrating and compromising, whereas focus on limitations is associated with maladaptive strategies such as avoiding and dominating. With age, older workers tend to experience more limitations and constraints and fewer opportunities in their career development, consequently resulting in a greater likelihood of utilizing maladaptive strategies to manage conflicts with their coworkers. Organizations are therefore recommended to offer older workers more opportunities to take part in training programs and get involved in the process of strategic planning and development in order to improve their perceived career opportunities, which will subsequently help to promote their use of adaptive strategies for handling workplace conflict.

### Limitations and Future Studies

Certain limitations should be acknowledged when interpreting the results obtained in this project. Firstly, both studies did not measure the perceived closeness with the conflict partner. According to SST, older adults show stronger preferences for emotionally close social partners (Fung et al., 1999) than younger adults. Older individuals may be less likely to use passive strategies when dealing with less close co-workers than when dealing with close co-workers. Future studies should examine the effect of emotional closeness on the use of conflict strategies.

Secondly, the two studies relied on the self-reported ratings to measure the use of various conflict strategies, which may be subject to recall and social desirability biases. Older adults’ positive memory bias (Mather and Carstensen, 2005) might influence them to report the conflicts that evoked more positive emotions, for example, the events in which they could handle satisfactorily to maintain interpersonal harmony. Thus, the inclusion of an objective measure of the conflict strategies, such as ratings from the opposing partner (e.g., Davis et al., 2009), could be considered in future research. Including a measure of social desirability scale is also recommended. Lastly, the indirect effects of age on the five conflict strategies through focus on opportunities and on limitations were replicated in both Studies 1 and 2. However, the cross-sectional data could not demonstrate the causal relationship between OFTP and conflict management. In the exploratory analyses, an alternate mediation model was tested, in which age was inputted as the independent variable, the five conflict strategies (integrating, compromising, obliging, avoiding, and dominating) as the mediators, and focus on opportunities and on limitations as the dependent variables. The mediation pathways of this alternate model were not statistically significant, except the indirect effect of age on focus on limitations through avoiding (*B* = −0.002, SE = 0.001, 95%CI [−0.004, −0.000]). As a result, future studies are recommended to experimentally manipulate working adults’ perception of opportunities and limitations since it will provide stronger evidence for the direct impacts of OFTP on conflict management.

## Conclusion

The findings from the two studies reveal that the age effects on five conflict strategies appeared to be more robust after incorporating the two dimensions of OFTP, focus on opportunities and on limitations, as mediators. When older workers perceive fewer opportunities at work, they are less likely to utilize constructive strategies, such as integrating and compromising, to manage their conflicts with co-workers. By contrast, when they perceive greater limitations and constraints, a greater tendency to use maladaptive strategies, such as avoiding, obliging, and dominating, is observed.

## Data Availability Statement

The raw data supporting the conclusions of this article will be made available by the authors, without undue reservation.

## Ethics Statement

The studies involving human participants were reviewed and approved by Human Subjects Ethics Sub-Committee, City University of Hong Kong. The patients/participants provided their written informed consent to participate in this study.

## Author Contributions

DY was responsible for funding acquisition, conceptualization, design and implementation of the studies, statistical analysis, and writing the manuscript. AH was responsible for conceptualization, statistical analysis, and writing the manuscript. Both authors contributed to the article and approved the submitted version.

## Conflict of Interest

The authors declare that the research was conducted in the absence of any commercial or financial relationships that could be construed as a potential conflict of interest.
